# A Short-Term Effect of Low-Dose Aspirin on Major Hemorrhagic Risks in Primary Prevention: A Case-Crossover Design

**DOI:** 10.1371/journal.pone.0098326

**Published:** 2014-05-30

**Authors:** I-Chen Wu, Ming-Yen Lin, Fang-Jung Yu, Hui-Min Hsieh, Kuei-Fen Chiu, Ming-Tsang Wu

**Affiliations:** 1 Kaohsiung Medical University, Kaohsiung, Taiwan; 2 Kaohsiung Medical University Hospital, Kaohsiung, Taiwan; 3 Kaohsiung Municipal Ta-Tung Hospital, Kaohsiung, Taiwan; 4 Kaohsiung Municipal Hsiao-Kang Hospital, Kaohsiung, Taiwan; Universidad de Valladolid, Spain

## Abstract

**Background:**

Very few studies have examined the risk of short-term adverse hemorrhage of low-dose aspirin use in primary prevention. This case-crossover study examined the transient effect of low-dose aspirin use on major hemorrhagic risks.

**Methods:**

A representative database of 1,000,000 patients randomly sampled from the Taiwan's National Health Insurance Research Database in 2000 was analyzed. The study cohort consisted of a total of 501,946 individuals, aged 30–95 years old, at risk of a major bleeding event in 2000. A case-crossover study was used to retrieve data on 10,905 incident patients with major hemorrhagic complications (3,781 cerebral and 7,124 gastrointestinal) and prescribed low-dose aspirin (≤300 mg/day) from 2000–2008. A 56-day time window (∼2 months) was used as the case period for which the odds ratio (OR) was estimated using the ratio of patients exposed during the 56-day case period only (1–56 days before the index date) compared to its corresponding 56-day control period only (57–112 days before the index date).

**Results:**

Four hundred eighty-nine (4.5%) of the 10,905 hemorrhagic patients had used low-dose aspirin during the 56-day case only period; 294 (2.7%) of the same patients had used low-dose aspirin during control only period. Low-dose aspirin use increase the risk of developing a major hemorrhage 1.33-fold (95% CI = 1.13–1.55, P<0.0001). Significance was found prominent in 4,453 non-hypertensive and non-diabetic subjects (Adjusted odds ratio  = 1.88, 95% CI = 1.21–2.91).

**Conclusion:**

Transient low-dose aspirin use increases risk for major hemorrhagic events in Han Chinese.

## Introduction

Long-term antiplatelet treatment has been estimated to reduce ∼25% of non-fatal myocardial infarction, non-fatal stroke, and vascular death [1]. Aspirin is an antiplatelet well-recognized in its use for the secondary prevention of cardiovascular events [1], [2], though its primary preventive benefit is limited by its adverse hemorrhagic effect [3]–[7]. In 2009, the Antithrombotic Trialists' (ATT) Collaboration study comprehensively reviewed and performed a meta-analysis of six primary prevention trials and sixteen secondary prevention trials [1]. The authors concluded that routine use of aspirin as primary prevention in those without previous diseases may be of questionable net benefit in the reduction of occlusive episodes because it increases the risk of major bleeding.

Because a more recent meta-analyses has revealed that aspirin has a chemopreventive effect on cancer incidence and mortality [8], [9], the question of whether prescribe aspirin should be prescribed as primary prevention agent or not has regained fresh interest [6], [7], [10]. This question may be more important for Asians, since, commented by Morimoto *et al*., the clinical guidelines of aspirin use in the primary prevention of cardiovascular events in Western countries may not be appropriately applied to Asian populations due to different disease preference in this population [3].

Recently, De Berardis *et al.*, analyzing a population-based cohort of 4.1 million Italian citizens, reported that low-dose daily aspirin use (≤300 mg) significantly increased the risk of both gastrointestinal and cerebral hemorrhages [11]. Although the finding was important, their subjects included those with or without previous hospitalization for cardiovascular events, making it difficult to determine aspirin-associated risk estimates of primary prevention only [2].

Using Taiwan's nationwide population-based insurance claims dataset, we performed a case-crossover study to investigate the impact of short-term (56 days) low-dose aspirin use on major bleeding events. This timeframe should make it possible to elucidate the transient effect of aspirin on the risk of acute events 12,13]. The use of case-crossover design may provide more reliable data because the same subjects, who are investigated at adjacent time points, serve as their own controls, minimizing the confounding of both known and unknown time-invariant variables between the study patients.

## Methods

This study tapped Taiwan's single-payer National Health Insurance (NHI), promulgated by the Taiwan government on March 01 1995 [14], [15]. After 1996, NHI claims data were digitalized and managed by Taiwan's National Health Research Institutes, creating a large medical claims database known as the National Health Insurance Research Database (NHIRD). As of 2007, 22.6 million of Taiwan's 23.0 million citizens were enrolled in Taiwan's NHI program, making the NHIRD one of the largest population-based insurance databases in the world [16].

### Data Sources

This study used a sampling cohort dataset obtained from NHIRD. National Health Research Institutes use a systematic sampling approach to randomly select a representative database of 1,000,000 patients from the year 2000 registry of all NHI enrollees (NHI 2000) [17]. We retrospectively and prospectively followed these patients from January 1, 1997 to December 31, 2008. This study was approved by Institutional Review Board of Kaohsiung Medical University Hospital. Because the patient identifiers in this national dataset were scrambled to the public for research purpose in Taiwan, the study was exempted from the requirement for written or verbal consents from patients. According to National Health Research Institutes, there are no significant differences in age, sex, or health care costs between the sampled group and all enrollees in NHI 2000 [16]. This dataset gives researchers access to comprehensive demographic data, including gender, date of birth, and income level as well as health care data, including date of admission or discharge, clinical diagnoses (up to five coexisting diagnoses listed on one claims record), medical procedures (up to five diagnostic or therapeutics procedures), expenditures, detailed drug prescriptions, and in-hospital deaths. NHI lists diagnoses using the *International Classification of Diseases, Ninth revision, clinical Modification (ICD-9-CM)* [18].

### Study Subjects

**Figure 1:**
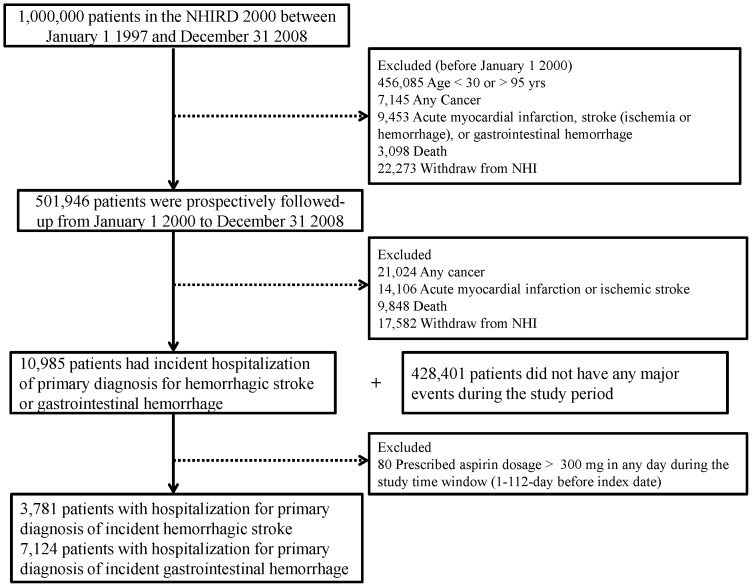
Study flowchart.

This case-crossover study recruited patients aged 30–95 years old in 2000 as potential study subjects (Figure 1). We excluded patients who had a previous claims record listing major cardiovascular or gastrointestinal problems as primary diagnosis as well as patients hospitalized with the primary diagnosis of cancer (*ICD-9-CM codes 140.xx-208.xx*), acute myocardial infarction (AMI) (*ICD-9-CM codes 410, 36.0, 36.1, 36.2, and 36.3*), ischemic stroke (*ICD-9-CM codes 433, 434, 436, 38.11, and 38.12*), major gastrointestinal hemorrhage (*ICD-9-CM codes 531.0, 531.2, 531.4, 531.6, 532.0, 532.2, 532.4, 532.6, 533.0, 533.2, 533.4, 533.6, 534.0, 534.2, 534.4, and 534.6*) or cerebral hemorrhage (*ICD-9-CM codes 430-432*) between January 1, 1997 and December 31, 1999. Patients expiring or leaving the NHI program for unknown reasons in 2000 were also excluded.

We prospectively followed the remaining relatively healthy patients starting in January 1 2000 until first primary diagnosis of AMI, ischemic stroke, major gastrointestinal hemorrhage, or cerebral hemorrhage during a hospital stay or death from other causes, withdrawal from the NHI program for unknown reasons, or the end of study period (December 31 2008). From those first diagnosed with major hemorrhagic complication, we excluded patients who were prescribed aspirin dosages >300 mg/day on any day during the study time period window (1–112 days before the date of that diagnosis), leaving us with only those prescribed low-dose aspirin (Figure 1).

### Data Collection of Study Subject Characteristics (Time-invariant Variables)

This study collected data on patient age, gender, income, place of insurance registry (Northern, Central, Southern, or Eastern), and urbanization level (rural area, satellite city, or urban). Diabetes (*ICD-9-CM code 250*) or hypertension (*ICD-9-CM codes 401–405*) was defined in a patient if he or she was diagnosed for the diseases in at least two outpatient claims or in one inpatient claim a year before the major hemorrhagic complication was first diagnosed. General health status was assessed by the Charlson co-morbidity index, which is the sum of the weighted score of 17 co-morbid conditions and is widely used to control confounding in epidemiological studies (Table S1) [19].

### Validation of Outcome and Exposure Variables

The database we tapped has been widely used for clinical epidemiological studies, and its disease diagnoses, drug prescription, and hospitalization data is reported to be of high quality by many studies [14], [20]–[23]. For example, 97.9% of patients with coded ischemic stroke in NHIRD were confirmed by radiological examination and clinical presentation [14]. In addition, the accuracy the record for aspirin prescribed was high in both first post-discharge visits (positive predictive value, PPV = 0.94) and during hospitalization (PPV = 0.88). For covariates, one previous study showed the claims dataset to have a high diagnostic accuracy of diabetes with sensitivity of 93.2% and PPV of 92.3% [20], [22].

### Case-Crossover Design

In a case-crossover design, each eligible study patient serves as his or her own control. The exposure of interest was low-dose aspirin (Anatomical Therapeutic Chemical code B01AC06). We estimated the odds ratio (OR) as ratio of patients exposed during the 56-day case period only (almost two months), defined as 1–56 days before the first diagnosis of major hemorrhagic complication, to the same patients exposed during the same 56-day control period only, defined as 57–112 days before first diagnosis of major hemorrhagic complication. The advantage of this case-crossover design approach is that the transient exposure effect on acute outcome can be examined without between-patient confounders, such as smoking, etc [12], [13], [15].

### Potential Time-variant Confounding Variables

To control for confounding variables, drugs that could potentially and significantly accelerate or reduce bleeding tendency and changes in their prescriptions over time were included in the model. These drugs were anticoagulants, antilipemic agents such as statin, nonsteroidal anti-inflammatory agents (NSAID), proton-pump inhibitors (PPI), antidepressants such as tricyclic antidepressants, selective serotonin reuptake inhibitors, monoamine oxidase inhibitors, or other antidepressants, and corticosteroid, all of which have been considered by a previous study [11]. Besides those aforementioned drugs, we also treated anti-hypertensive agents, including angiotensin converting enzyme inhibitors, angiotensin receptor blockers, α-blockers, β-blockers, calcium channel blockers, diuretics, central α_2_ agonist, and vasodilators, as another time-variant variable. Exposure to these drugs was defined as having a prescription of one of them at least one day during the case period (56 days before first diagnosis of major hemorrhagic complication) or control period (57–112 days before first diagnosis of major hemorrhagic complication). In addition, the number of outpatient visits during these two time periods was counted.

### Statistical Analyses

For the case-crossover analyses, we first used McNemar test to investigate the significance of aspirin use between case and control period, and conditional logistic regression to estimate the odds ratio (OR) and its 95% confident interval (CI) of gastrointestinal or cerebral hemorrhage or both. Then, subgroup analyses were performed by stratifying the different time-invariant and time-variant characteristics of the patients, including age, gender, place of insurance registry, urbanization, year and season of index date and other medical conditions diseases such as Charlson co-morbidity index, hypertension, and diabetes as well as the presence of other confounding drugs during the study time window.

Conditional logistic regressions were performed to explore the effect of aspirin use on hemorrhagic events after adjusting for the time-variant confounding factors which were significant in the univariate analysis listed in Table 1 and were considered by the previous study [11], in total and in different subgroup analyses of time-invariant variables. We treated hypertension either the time-invariant variable (history of hypertension) or the time-variant variable (anti-hypertensive agents) in the regression models and found the effect of low-dose aspirin did not change dramatically. Thus, in the rest of the analyses, we treated hypertension as the time-invariant variable. We also categorized the study patients by history of hypertension and diabetes to examine the magnitude of low-dose aspirin risk.

**Table 1:** Demographics and Clinical Characteristics of 10,905 Study Patients with Incident Major Gastrointestinal or Cerebral Hemorrhagic Event, 2000-2008.

Characteristics	N (%) or Mean ± SD
Age groups (years)	
30–64	5,528 (50.7)
≥65	5,377 (49.3)
Male	6,967 (63.9)
Major event	
Gastrointestinal hemorrhage	7,124 (65.3)
Cerebral hemorrhage	3,781 (34.7)
Geographical area	
Northern	4,724 (43.3)
Central	2,571 (23.6)
Southern	3,159 (29.0)
Eastern	451 (4.1)
Urbanization	
Rural area or satellite city	3,408 (31.3)
Urban	7,497 (68.7)
Hypertension	
No	5,126 (47.0)
Yes	5,779 (53.0)
Diabetes	
No	8,532 (78.2)
Yes	2,373 (21.8)
Charlson index score	
<3	7,124 (65.3)
≥3	3,781 (34.7)
Year of event	
2000–2004	6,618 (60.7)
2005–2008	4,287 (39.3)
Season of event	
JAN-MAR	3,043 (27.9)
APR-JUN	2,624 (24.1)
JUL-SEP	2,430 (22.2)
OCT-DEC	2,808 (25.8)
**Case period of time variant variables (1–56 days)** a	
Use of anticoagulants	150 (1.4)
Use of antilipemic agents	581 (5.3)
Use of nosteroidal anti-inflammatory agents	4,626 (42.4)
Use of proton pump inhibitors	667 (6.1)
Use of antidepressants	477 (4.4)
Use of corticosteroids	899 (8.2)
Number of outpatient visits	4.73±4.48
**Control period of time variant variables (57–112 days)** a	
Use of anticoagulants	121 (1.1)
Use of antilipemic agents	522 (4.8)
Use of nosteroidal anti-inflammatory agents	3,396 (31.1)
Use of proton pump inhibitors	2,28 (2.1)
Use of antidepressants	437 (4.0)
Use of corticosteroids	736 (6.8)
Number of outpatient visits	8.44±7.86

Abbreviation: SD =  standard deviation.

a The difference of two periods was compared by paired t test or McNemar test whichever appropriate: use of anticoagulants, p value  = 0.0021; use of antilipemic agents, p value  = 0.0028; use of nosteroidal anti-inflammatory agents, p value <0.0001; use of proton pump inhibitors, p value <0.0001; use of antidepressants, p value  = 0.0305; use of corticosteroids, p value <0.0001; and number of outpatient visits, p value <0.0001.

In addition to our analysis of a 56-day case period, we also computed odds ratios for different case periods, including 28-, 84-, 112-, 140-, 168-, 252-, and 336-day durations (4-, 12-, 16-, 20-, 24-, 36-, and 48-week durations, respectively) to test the robustness of the results. This kind of analysis was also applied to different sites of hemorrhagic complications. All statistical operations were performed using SAS 9.2 statistical software; two-sided P value <0.05 was considered significant.

## Results

A total of 10,985 cohort patients were hospitalized for a primary diagnosis of incident hemorrhagic complication between 2000 and 2008 (Figure 1). We excluded 80 patients who were prescribed aspirin >300 mg on any one day during the study period (1 to112-days before index date). The remaining 10,905 (3,781 with cerebral hemorrhages and 7,124 with gastrointestinal hemorrhages) were included in our final analysis (Table 1). The mean (standard deviation, SD) age of these patients was 62.2 (15.1) years. The hemorrhagic event most often occurred in men (63.9%), urban areas (69.1%), patients with a history of hypertension (53.0%), and diabetes-free patients (78.2%).The uses of anti-hypertensive agents in the case period and control period were 12.5% (n = 1,361) and 6.8% (n = 743), respectively. The mean number (SD) of outpatient visits was 4.72 (4.48) during the 1 to 56-day case period and 8.44 (7.86) in the 57 to 112-day control period (P<0.0001) (Table 1).

Of the 10,905 patients, 489 (4.5%) had used low-dose aspirin during the 56-day case only period and 294 (2.7%) during the control only period (Table 2). User rates of aspirin were 12.8% (n = 1,393) in the 1 to 56-day case period and 11.0% (n = 1,198) in the 57- to 112-day control period. A total of 1,687 patients used aspirin over the two 56-day periods (1- to 112-day); in this group, the daily averaged ± SD dosage of aspirin was 54.4±34.2 mg/day (median dosage, 55.4 mg/day; interquartile range (IQR), 25.0–83.0 mg/day). Only 99 study patients were, on average, prescribed more than 100 mg per day in this 1–112-day time window, the maximum average prescribed dose being 213.4 mg/day.

**Table 2:** Low-dose Aspirin Use between the Case (1–56 days) and Control Periods (57–112 days) for Major Hemorrhagic Risks by Patient Characteristics.

Variables	Use only in the case period	Use only in the control period	Use in both periods	Nonuse in both periods	Crude odds ratio^a^	95% CI^a^	P value	Adjusted odds ratio^b^	95% CI	P value
All patients (N = 10,905)	489	294	904	9,218	1.66	1.44–1.92	<0.0001	1.33	1.13–1.55	<0.0001
Age group (years)										
30–64 (N = 5,528)	159	96	243	5,030	1.66	1.29–2.13	<0.0001	1.34	1.02–1.76	0.0374
≥65 (N = 5,377)	330	198	661	4,188	1.67	1.40–1.99	<0.0001	1.32	1.09–1.60	0.0049
Sex										
Female (N = 3,938)	188	114	379	3,257	1.65	1.31–2.08	<0.0001	1.32	1.04–1.71	0.0252
Male (N = 6,967)	301	180	525	5,961	1.67	1.39–2.01	<0.0001	1.32	1.08–1.62	0.0067
Hemorrhagic site										
Gastrointestinal hemorrhage (N = 7,124)	339	193	668	5,924	1.76	1.47–2.10	<0.0001	1.35	1.10–1.65	0.0029
Cerebral hemorrhage (N = 3,781)	150	101	236	3,294	1.49	1.15–1.91	0.0020	1.28	0.99–1.66	0.0623
Places of insurance registry										
Northern (N = 4,724)	201	127	422	3,974	1.58	1.27–1.98	<0.0001	1.23	0.96–1.57	0.1052
Central (N = 2,571)	136	72	215	2,148	1.89	1.42–2.51	<0.0001	1.48	1.09–2.01	0.0119
Southern (N = 3,159)	132	77	226	2,724	1.71	1.29–2.27	0.0001	1.49	1.10–2.02	0.0104
Eastern (N = 451)	20	18	41	372	1.11	0.59–2.10	0.7456	0.82	0.41–1.62	0.5655
Urbanization levels										
Rural area or satellite city (N = 3,408)	154	93	250	2,911	1.66	1.28–2.14	0.0001	1.27	0.96–1.68	0.0892
Urban (N = 7,497)	335	201	654	6,307	1.67	1.40–1.99	<0.0001	1.35	1.12–1.64	0.0019
Hypertension										
No (N = 5,126)	120	49	139	4,818	2.45	1.76–3.41	<0.0001	1.93	1.34–2.78	0.0004
Yes (N = 5,779)	369	245	765	4,400	1.51	1.28–1.77	<0.0001	1.23	1.04–1.47	0.0189
Diabetes										
No (N = 8,532)	334	194	501	7,503	1.72	1.44–2.06	<0.0001	1.33	1.09–1.61	0.0044
Yes (N = 2,373)	155	100	403	1,715	1.55	1.21–1.99	0.0006	1.31	1.00–1.71	0.0481
Charlson index score										
<3 (N = 7,124)	247	145	405	6,327	1.70	1.39–2.09	<0.0001	1.34	1.07–1.68	0.0105
≥3 (N = 3,781)	242	149	499	2,891	1.62	1.32–1.99	<0.0001	1.34	1.08–1.67	0.0086
Year of event										
2000–2004 (N = 6,618)	257	159	486	5,716	1.62	1.33–1.97	<0.0001	1.29	1.04–1.60	<0.0001
2005–2008 (N = 4,287)	232	135	418	3,502	1.72	1.39–2.13	<0.0001	1.37	1.09–1.73	0.0081
Season of event										
JAN-MAR (N = 3,043)	118	76	228	2,621	1.55	1.16–2.07	0.0026	1.29	0.95–1.75	0.1098
APR-JUN (N = 2,624)	115	81	244	2,184	1.42	1.07–1.89	0.0152	1.09	0.80–1.48	0.5950
JUL-SEP (N = 2,430)	125	62	190	2,053	2.02	1.49–2.73	<0.0001	1.54	1.10–2.15	0.0114
OCT-DEC (N = 2,808)	131	75	242	2,360	1.75	1.32–2.32	<0.0001	1.40	1.02–1.92	0.0356
**Use of confounding medicine (Time-variant variables)** c										
Anticoagulants										
No (N = 10,725)	472	286	880	9,087	1.65	1.43–1.91	<0.0001	1.31	1.12–1.54	0.0008
Yes (N = 180)	17	8	24	131	2.13	0.92–4.92	0.0179	1.95	0.81–4.70	0.1355
Antilipemic agents										
No (N = 10,158)	410	245	718	8,785	1.67	1.43–1.96	<0.0001	1.34	1.13–1.59	0.0008
Yes (N = 747)	79	49	186	433	1.61	1.13–2.30	0.008	1.39	0.94–2.04	0.0958
Nosteroidal anti-inflammatory agents										
No (N = 5,300)	204	106	357	4,633	1.93	1.52–2.43	<0.0001	1.50	1.17–1.93	0.0013
Yes (N = 5,605)	285	188	547	4,585	1.52	1.26–1.82	<0.0001	1.20	0.98–1.47	0.0840
Proton pump inhibitors										
No (N = 10,110)	448	264	844	8,554	1.70	1.46–1.98	<0.0001	1.33	1.13–1.57	0.0006
Yes (N = 795)	41	30	60	664	1.37	0.85–2.19	0.1917	1.17	0.70–1.97	0.5496
Antidepressants										
No (N = 10,277)	451	259	806	8,761	1.74	1.50–2.03	<0.0001	1.38	1.16–1.62	0.0002
Yes (N = 628)	38	35	98	457	1.09	0.69–1.72	0.7255	0.92	0.56–1.50	0.7279
Corticosteroids										
No (N = 9,615)	410	254	788	8,163	1.61	1.38–1.89	<0.0001	1.30	1.11–1.54	0.0024
Yes (N = 1,290)	79	40	116	1,055	1.98	1.35–2.89	0.0004	1.45	0.96–2.19	0.0796

a Calculated by univariate conditional logistic regression and tested by McNemar's test.

b Calculated by multivariate conditional logistic regression after adjusting for time-variant variables and number of outpatient visits.

c Calculated by multivariate conditional logistic regression after adjusting for other time-variant variables and number of outpatient visits.

After adjusting for other time-variant medication variables and number of outpatient visits, we found that low-dose aspirin use conferred a 1.33-fold risk (95% CI = 1.13–1.55, P<0.0001) of major hemorrhage during the case period, compared to the control period. Even after excluding the 99 study patients whose averaged daily dosage was >100 mg/day, the result remained similar, with an adjusted odds ratio (AOR) of 1.36 (95% CI = 1.17–1.61, P<0.0001). Replacing history of hypertension by anti-hypertensive agents and adjusting this time-variant variable in the regression model, we found that low-dose aspirin use conferred a 1.18-fold risk (95% CI = 1.004–1.39, P = 0.0443) of major hemorrhage during the case period, compared to the control period. Similar and significantly increased risks related to low-dose aspirin use were also found in almost all other sub-categories of the time-invariant variables, including age, gender, urbanization level, hypertension, diabetes, Charlson index score, and year of event (Table 2). Because most patients were not prescribed other study drugs, the significant risks of low-dose aspirin use on bleeding events were consistently present in non-user groups (Table 2).

**Figure 2:**
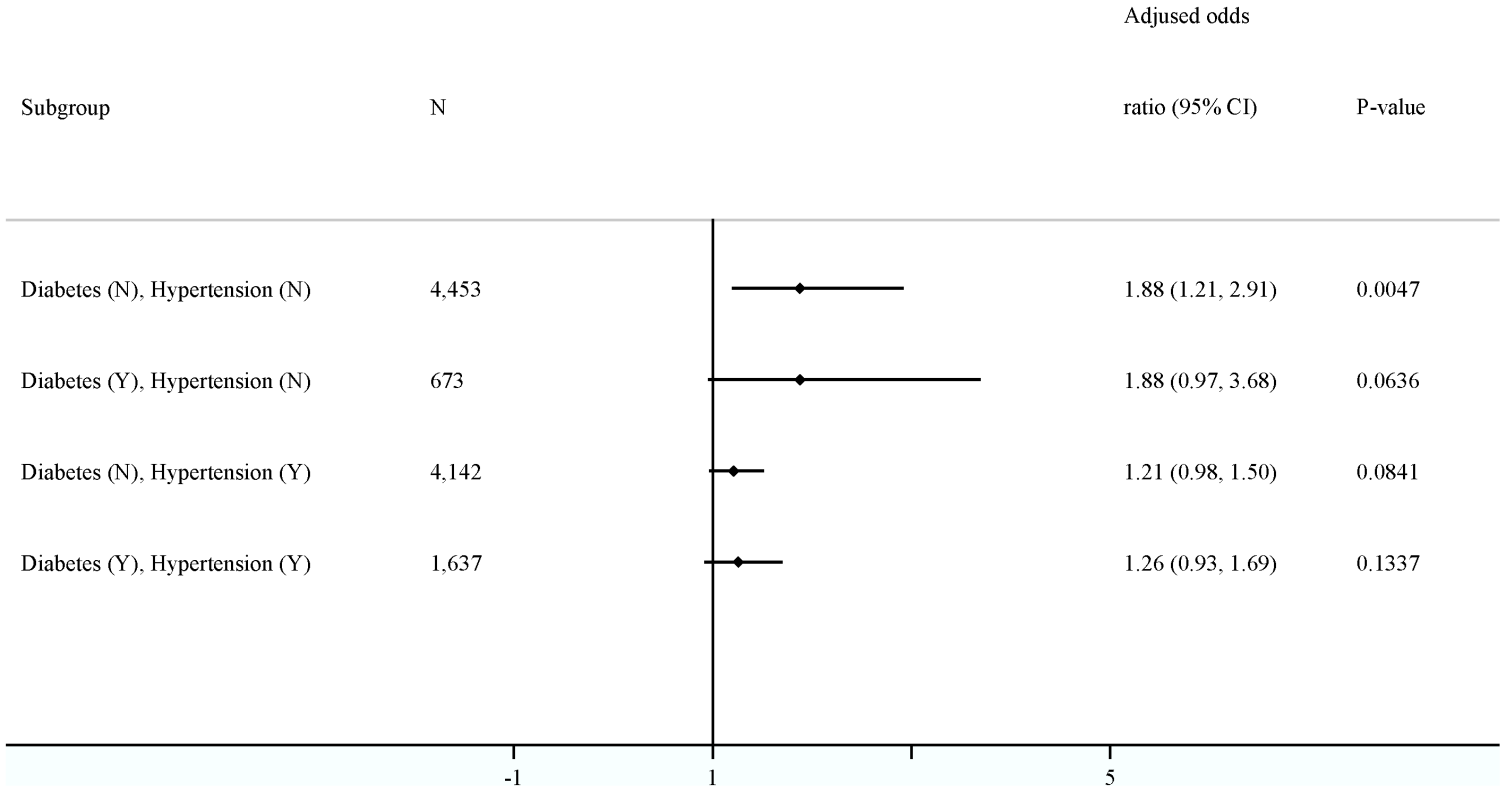
Subgroups analyses categorized by diabetes and hypertension in a 56-day case period of time window.

The AOR risk of bleeding events associated with aspirin use was 1.35 (95% CI = 1.10–1.65, P = 0.0029) in gastrointestinal hemorrhage and 1.28 (95% CI = 0.99–1.66, P = 0.0623) in cerebral hemorrhage (Table 2). Figure 2 shows increased risks range from 1.21- to 1.88-fold when further categorized by diabetes and hypertension. This association reached significance in the subgroup of subjects without a history of having both diabetes and hypertension (AOR = 1.88, 95% CI = 1.21–2.91, P = 0.0047). When categorized by anti-hypertensive agents (time-variant variable), the hemorrhagic risk was similar in subjects with (n = 5,014) and without (n = 5,891) the prescription of antihypertensive agents in the observational period (AOR = 1.32, 95% CI = 1.11–1.56, P = 0.0017 for subjects with use of antihypertensive agents; AOR = 1.42, 95% CI = 0.96–2.10, P = 0.0766 for subjects without use of antihypertensive agents).

As can be seen in Table 3, we analyzed our data using different case and control time windows, with one window being as long as almost a year. We found that exposure to aspirin had a similar and significant effect on hemorrhagic events during all time windows, except for the 28-day time window (Table 3). The results were consistently significant in those with gastrointestinal hemorrhages (Table S2).

**Table 3:** Low-dose Aspirin and Major Hemorrhagic Risks in Different Time Windows.

Case period of time window (Days)^a^	Use only in the case period	Use only in the control period	Use in both periods	Nonuse in both periods	Crude odds ratio	95% CI	P value	Adjusted odds ratio^b^	95% CI	P value
28	437	344	612	9,512	1.27	1.10–1.46	0.0009	1.04	0.89–1.21	0.6491
56	489	294	904	9,218	1.66	1.44–1.92	<0.0001	1.33	1.13–1.55	<0.0001
84	461	236	1,125	9,065	1.95	1.67–2.29	<0.0001	1.61	1.36–1.90	<.0.0001
112	505	271	1,170	8,922	1.86	1.61–2.16	<0.0001	1.57	1.35–1.84	<.0.0001
140	557	329	1,185	8,778	1.69	1.48–1.94	<0.0001	1.45	1.25–1.67	<.0.0001
168	598	368	1,210	8,656	1.63	1.43–1.85	<0.0001	1.43	1.25–1.63	<.0.0001
252	764	419	1,228	8,364	1.82	1.62–2.05	<0.0001	1.59	1.41–1.81	<0.0001
336	889	444	1,263	8,125	2.00	1.79–2.24	<0.0001	1.69	1.50–1.91	<0.0001

a Additional 18, 37, 56, 73, 130, and 184 study subjects were excluded from time windows in 84, 112, 140, 168, 252, and 336 days of case period, respectively, due to the prescription dosage of aspirin >300 mg in any day of the study period (case + control periods).

b After adjusting for time-variant medication variables and number of outpatient visits.

## Discussion

In this case-crossover study using a nationwide representative sampling cohort, we found that short-time low-dose aspirin use increased the risk of major bleeding events among relatively healthy patients. Its adverse health effect was found to be particularly prominent in gastrointestinal hemorrhages and was consistently present when it was prescribed for two months or more. While increased risk was found in patients with and without diabetes as well as patients with and without hypertension, the risk was found to be higher in patients without these diseases. The case-crossover design of the study eliminated confounding by between-subject time-invariant factors and reduced the likelihood of “reverse causation” often associated with retrospective cohort designs [12], [13]. In addition, the robustness of these results was confirmed by analyzing the data using different time windows up to a year.

The 33% increased risk we found was slightly lower than that reported by De Berardis *et al.*, who reported a ∼55% increased risk [11]. This difference was probably due to the difference in study designs. Their controls may not have received aspirin, making it possible that they were healthier than the cases with whom they were matched. This might have led to an overestimation of the risk. In the current case-crossover study, the same patient was followed over two-adjacent time periods, a case period and control period. Thus, differences in the health status of our subjects were not so much an issue.

This study found the increased risk of major bleeding to be similar for patients with and without diabetes (31% *vs.* 33% increase, respectively). This finding was slightly different from De Berardis *et al.*, who reported a large and significant increase in such risk in patients who did not have diabetes but only small and insignificant increase in patients with diabetes (66% *vs.* 9% increase, respectively), again probably due to different study designs [11]. De Berardis *et al.* also included case patients with previous hospitalization for cardiovascular events. We found increased risk of bleeding in both non-hypertensive (93%) and hypertensive subjects (23%). Together, our findings raise concern regarding the use of low-dose aspirin for primary prevention of cardiovascular diseases in the Taiwanese population with diabetes or hypertension.

In the subgroup analyses of time-variant variables, we found that the hemorrhagic risk was slightly higher in the non-user group of NSAID (AOR = 1.50) than in the user group of NSAID (AOR = 1.20), suggesting whether use of NSAID in the observational period can modify the effect of low-dose aspirin use on the hemorrhagic event (Table 2). The similar findings were also noted in the groups of PPI use.

There is a wide reported variation in response to antiplatelet treatment in different ethnic groups (0.4 to 35%) [24]–[26]. While a high response to aspirin might represent good prognosis in patients undergoing antiplatelet treatment, it may also point to greater risk of bleeding events in the same patients [3], [24]. In Japan, Morimoto *et al.*, using existing published data on the rates of coronary heart disease, hemorrhagic stroke, and major gastrointestinal bleeding from the Japanese population, found that the benefit of aspirin use only outweighed risk in subjects older than 40 years who had both diabetes and hypertension [3]. Although we did not estimate risk-and-benefit ratio, we found increased risk of aspirin-associated bleeding in subjects with and without diabetes or hypertension, with the most significant risk found in those who did not have these diseases (Figure 2).

The mechanisms underlying the first occurrence of occlusive vascular diseases and recurrence may be different, suggesting that the benefit of aspirin in the secondary prevention of vascular diseases may not be extrapolated to its use in primary prevention. Aspirin is a non-selective cyclo-oxygenase inhibitor; its half-life is approximately 6 hours in plasma. Although the half-life of aspirin is short, to generate the health effect from reducing the synthesis of prostaglandins in the vascular wall resulting in constricting the vessel wall and enhancing platelet adhesion to the vessel wall in healthy people takes much longer than the original exposure half-life of aspirin. The present study in Table 3 shows that hemorrhagic events significantly increase after 56-day time window of aspirin use, but not before 28-day time window, suggesting the health adverse effect of low-dose aspirin use needs to take action longer than the exposure half-life of aspirin in humans.

The advocacy of low-dose aspirin use for primary prevention has regained some momentum because it has recently been reported to protect against the development of some forms of cancer [9], [27], [28]. One recent comprehensive meta-analysis analyzed the time course of risks and benefits of aspirin use in 51 randomized controlled trials [9], including six clinical trial studies of the daily use of aspirin as primary prevention [29]–[34]. That study found major extracranial bleeding significantly to increase 1.95-fold (95% CI = 1.47–2.59) during the first three years of aspirin use, though major vascular events decreased 0.82-fold (95% CI = 0.72–0.90). They also reported that it took three years until aspirin's protection against cancer would be evident (aspirin group *vs.* control, OR = 0.70, 95% CI = 0.67–0.98). These two findings suggest that subjects may need to avoid the risk of major bleeding complications during the first three years of aspirin use to enjoy the benefit of cancer prevention. That study, however, only addressed low-dose aspirin associated extracranial bleeding [9], not intracranial hemorrhages, which is more common among Asians. That meta-analysis did not include some other primary prevention clinical trials, such as the Women's Health Study and Physicians' Health Study, which studied alternate-day aspirin use [35], [36].

When attempting to prevent cardiovascular disease, physicians may need to be reminded that treatment with antiplatelets, including low-dose aspirin, is not the sole choice for primary prevention of cardiovascular diseases. Many other alternative preventive strategies of lifestyle changes such as regular exercise, weight loss, saturated-fat diet control, smoking cessation and sugar restriction can be used first in primary prevention to avoid unintentional harm by antiplatelet agents [37]–[40].

This study has several limitations. One limitation is that our study findings could be influenced by time-variant variables [15], for example, abrupt emotional distress, though this confounding can be reduced in part by controlling for antidepressants. However, if the time-variant variables were caffeine-containing medicines, sexual activity, and physical exercise, etc. which could independently trigger intracranial hemorrhages and were not available in our study [40], the confounding bias was still likely. Another limitation is that aspirin exposure was based on prescription information only, and thus we cannot know whether the study patients actually took the drug as prescribed. This bias is likely to cause random misclassification of exposure and underestimates of our findings. Similarly, aspirin can be purchased easily over-the-counter, though such purchases are reduced by a national insurance system that allows patients to see almost any physician they want and covers most drug prescriptions, including aspirin. Another limitation is that we did not take into account several important lifestyle risk factors of major bleeding such as obesity, cigarette smoking, or alcohol drinking because that data was not available in this study cohort [17]. However, because this is a case-crossover study, this bias was likely to be trivial. By contrast, our study design cannot completely eliminate the bias of confounding by indication, if patients with cardiovascular risk factors prescribed low-dose aspirin had also commonly to have higher bleeding tendency in intracranial site. Again, we expect this bias is minimial, because the indication for low-dose aspirine use should not vary dramatically in a short observed period (around 4 months). Still another limitation is that we studied a population largely consisting of people Han Chinese descent, so our results may not generalized non-Asians.

In conclusion, this study shows that low-dose aspirin use increased acute major bleeding events, particularly gastrointestinal bleeding. While this increased risk was found in both diabetes and non-diabetes patients as well as both hypertension and non-hypertension patients, it was more prominent among patients without diabetes and hypertension. Further large prospective studies are warranted to investigate the risks and benefits of low-dose aspirin use in primary prevention to help physicians make appropriate recommendations for patients without previous history of cardiovascular or cerebrovascular diseases.

## Supporting Information

Table S1 ICD-9-CM Diagnosis Codes for Constructing Deyo-Charlson Comobidity Index.^a^
(DOCX)

Table S2 Usage of Confounding Medicine Between the Case (1-56-days) and Control Periods (57-112-days) for Major Hemorrhagic Risks by Hemorrhagic Types.
(DOCX)
