# Supplementary material for: A Short-Term Effect of Low-Dose Aspirin on Major Hemorrhagic Risks in Primary Prevention: A Case-Crossover Design
Source: PLoS One. 2014 May 30;9(5):e98326. doi: 10.1371/journal.pone.0098326 (PMC4039487; doi:10.1371/journal.pone.0098326)
Supplement: Table S1 — ICD-9-CM Diagnosis Codes for Constructing Deyo-Charlson Comobidity Index.a (DOCX) [file pone.0098326.s001.docx]

**Table S1. ICD-9-CM Diagnosis Codes for Constructing Deyo-Charlson Comobidity Index^a^**

| **Weights** | **Condition** | **ICD-9 Diagnosis Codes** |
| --- | --- | --- |
| 1 | Myocardial infarction | 410.xx, 412.xx |
|  | Congestive heart failure | 398.91, 402.01, 402.11, 402.91, 404.01, 404.03, 404.11, 404.13, 404.91, 404.93, 425.4, 425.5, 425.7, 425.8, 425.9, 428 |
|  | Peripheral vascular disease | 093.0, 437.3, 440, 441, 443.1, 443.2, 443.8, 443.9, 447.1, 557.1, 557.9, V434 |
|  | Cerebrovascular disease | 362.34, 430, 431, 432, 433, 434 , 435, 436, 437, 438 |
|  | Dementia | 290, 294.1, 331.2 |
|  | Chronic pulmonary disease | 416.8, 416.9, 490, 491, 492, 493, 494, 495, 496, 500, 501, 502, 503, 504, 505, 506.4, 508.1, 508.8 |
|  | Rheumatic disease | 446.5, 710.0, 710.1, 710.2, 710.3, 710.4, 714.0, 714.1, 714.2, 714.8, 725 |
|  | Peptic ulcer disease | 531, 532, 533, 534 |
|  | Mild liver disease | 070.22, 070.23, 070.32, 070.33, 070.44, 070.54, 070.6, 070.9, 570, 571, 573.3, 573.4, 573.8, 573.9,V42.7 |
|  | Diabetes without chronic complication | 250.0, 250.1, 250.2, 250.3, 250.8, 250.9 |
| 2 | Hemiplegia or paraplegia | 334.1, 342, 343, 344.0, 344.1, 344.2, 344.3, 344.4, 344.5, 344.6, 344.9 |
|  | Renal disease | 403.01,403.11,403.91,404.02,404.03,404.12,404.13,404.92,404.93,582, 583.0, 583.1, 583.2, 583.4, 583.6, 583.7, 585, 586, 588.0,V42.0,V45.1,V56 |
|  | Diabetes with chronic complication | 250.4, 250.5, 250.6, 250.7 |
|  | Any malignancy, including lymphoma and leukemia, except malignant neoplasm of skin | 140, 141, 142, 143, 144, 145, 146, 147, 148, 149, 150, 151, 152, 153, 154, 155, 156, 157, 158, 159, 160, 161, 162, 163, 164, 165, 170, 171, 172, 174, 175, 176, 179, 180, 181, 182, 183, 184, 185, 186, 187, 188, 189, 190, 191, 192, 193, 194, 195, 200, 201, 202, 203, 204, 205, 206, 207, 208, 238.6 |
| 3 | Moderate to severe liver disease | 456.0, 456.1, 456.2, 572.2, 572.3, 572.4, 572.8 |
| 6 | Metastatic solid tumor | 196, 197, 198, 199 |
|  | AIDS | 042, 043, 044 |

^a^Reference 19.
