# Supplementary material for: A Short-Term Effect of Low-Dose Aspirin on Major Hemorrhagic Risks in Primary Prevention: A Case-Crossover Design
Source: PLoS One. 2014 May 30;9(5):e98326. doi: 10.1371/journal.pone.0098326 (PMC4039487; doi:10.1371/journal.pone.0098326)
Supplement: Table S2 — Usage of Confounding Medicine Between the Case (1-56-days) and Control Periods (57-112-days) for Major Hemorrhagic Risks by Hemorrhagic Types. (DOCX) [file pone.0098326.s002.docx]

**Table S2. Usage of Confounding Medicine Between the Case (1-56-days) and Control Periods (57-112-days) for Major Hemorrhagic Risks by Hemorrhagic Types.**

| **Case period of time window (Days)** | | **Use only in the case period** | **Use only in the control period** | **Use in both periods** | **Nonuse in both periods** | **Crude odds ratio** | **95% CI** | **P value** | **Adjusted odds ratio^a^** | **95% CI** | **P value** |
| --- | --- | --- | --- | --- | --- | --- | --- | --- | --- | --- | --- |
| **Gastrointestinal hemorrhage** | |  |  |  |  |  |  |  |  |  |  |
| 28 (N=7,124) | | 316 | 238 | 453 | 6,117 | 1.33 | 1.12-1.57 | 0.001 | 1.07 | 0.88-1.30 | 0.4884 |
| 56 (N=7,124) | | 339 | 193 | 668 | 5,924 | 1.76 | 1.47-2.10 | <0.0001 | 1.35 | 1.10-1.65 | 0.0029 |
| 84 (N=7,111) | | 318 | 142 | 819 | 5,832 | 2.24 | 1.84-2.73 | <0.0001 | 1.83 | 1.48-2.27 | <0.0001 |
| 112 (N=7,100) | | 347 | 163 | 843 | 5,747 | 2.13 | 1.77-2.56 | <0.0001 | 1.80 | 1.47-2.20 | <0.0001 |
| 140 (N=7,089) | | 373 | 188 | 856 | 5,672 | 1.98 | 1.67-2.36 | <0.0001 | 1.69 | 1.40-2.05 | <0.0001 |
| 168 (N=7,075) | | 402 | 209 | 867 | 5,597 | 1.92 | 1.63-2.27 | <0.0001 | 1.68 | 1.41-2.01 | <0.0001 |
| 252 (N=7,031) | | 515 | 249 | 862 | 5,405 | 2.07 | 1.78-2.41 | <0.0001 | 1.80 | 1.53-2.11 | <0.0001 |
| 336 (N=6,993) | | 606 | 284 | 854 | 5,249 | 2.13 | 1.85-2.46 | <0.0001 | 1.77 | 1.52-2.06 | <0.0001 |
| **Cerebral hemorrhage** | |  |  |  |  |  |  |  |  |  |  |
| 28 (N=3,781) | | 121 | 106 | 159 | 3,395 | 1.14 | 0.88-1.48 | 0.3195 | 0.97 | 0.74-1.27 | 0.7977 |
| 56 (N=3,781) | | 150 | 101 | 236 | 3,294 | 1.49 | 1.15-1.91 | 0.0020 | 1.28 | 0.99-1.66 | 0.0623 |
| 84 (N=3,776) | | 143 | 94 | 306 | 3,233 | 1.52 | 1.17-1.97 | 0.0015 | 1.33 | 1.01-1.73 | 0.0390 |
| 112 (N=3,768) | | 158 | 108 | 327 | 3,175 | 1.46 | 1.15-1.87 | 0.0023 | 1.30 | 1.01-1.67 | 0.0439 |
| 140 (N=3,760) | | 184 | 141 | 329 | 3,106 | 1.31 | 1.05-1.63 | 0.0171 | 1.16 | 0.93-1.46 | 0.1877 |
| 168 (N=3,757) | | 196 | 159 | 343 | 3,059 | 1.23 | 1.00-1.52 | 0.0496 | 1.13 | 0.91-1.40 | 0.2677 |
| 252 (N=3,744) | | 249 | 170 | 366 | 2,959 | 1.47 | 1.21-1.78 | 0.0001 | 1.36 | 1.12-1.66 | 0.0022 |
| 336 (N=3,728) | | 283 | 160 | 409 | 2,876 | 1.77 | 1.46-2.15 | <0.0001 | 1.60 | 1.31-1.95 | <0.0001 |
| ^a^After adjusting for aspirin use, the other time-variant medication variables, and number of outpatient visits | | | | | | | | | | | |
|  | | | | | | | | | | | |
